# From Africa to Europe: evidence of transmission of a tropical *Plasmodium* lineage in Spanish populations of house sparrows

**DOI:** 10.1186/s13071-019-3804-1

**Published:** 2019-11-21

**Authors:** Martina Ferraguti, Josué Martínez-de la Puente, Luz García-Longoria, Ramón Soriguer, Jordi Figuerola, Alfonso Marzal

**Affiliations:** 1 Present Address: Department of Anatomy, Cellular Biology and Zoology, University of Extremadura (UEx), Badajoz, Spain; 2 Estación Biológica de Doñana (EBD-CSIC), Seville, Spain; 3 Centro de Investigación Biomédica en Red de Epidemiología y Salud Pública (CIBERESP), Madrid, Spain; 4 Department of Biology, Molecular Ecology and Evolution Lab, Ecology Building, Lund University, 22362 Lund, Sweden

**Keywords:** Avian malaria parasites, Haemosporidia, Geographical range shift, PAGRI02, *Passer domesticus*, Wild birds

## Abstract

**Background:**

Avian malaria parasites are a highly diverse group that commonly infect birds and have deleterious effects on their hosts. Some parasite lineages are geographically widespread and infect many host species in many regions. Bird migration, natural dispersal, invasive species and human-mediated introductions into areas where competent insect vectors are present, are probably the main drivers of the current distribution of avian malaria parasites.

**Methods:**

A total of 412 and 2588 wild house sparrows (*Passer domesticus*) were captured in 2012 and 2013 in two areas of the Iberian Peninsula (central and southern Spain, respectively). Genomic DNA was extracted from blood samples; parasite lineages were sequenced and identified by comparing with GenBank and/or MalAvi databases.

**Results:**

Thirteen *Plasmodium* lineages were identified in house sparrows corresponding to three major clades. Five individuals were infected by the African *Plasmodium* lineage PAGRI02, which has been proposed to actively circulate only in Africa.

**Conclusions:**

Despite the low prevalence of PAGRI02 in sparrows in Spain, our results suggest that the area of transmission of this parasite is more widespread than previously thought and covers both Africa and Europe. Further studies of the global distribution of *Plasmodium* lineages infecting wild birds are required to identify the current transmission areas of these parasites. This is vital given the current scenario of global change that is providing new opportunities for avian malaria transmission into areas where parasites were previously absent.

## Background

Avian malaria and related haemosporidian parasites are a highly diverse group of organisms that commonly infect birds. Haemosporidians belonging to the genus *Plasmodium* are widespread vector-borne protozoans that naturally infect birds on all continents except Antarctica [1]. Species of this parasite genus harm vertebrate hosts by reducing their reproductive success [2, 3] and the survival rates of infected individuals [4–6].

The use of molecular techniques to study avian malaria parasites has resulted in identification of a great diversity of lineages infecting birds worldwide [7]. To date, 3571 haemosporidian lineages infecting 1749 bird species have been recorded (MalAvi database version 2.4.0, accessed August 2019 [7]). Some parasite lineages are geographically widespread and infect many host species in many different regions. For instance, the widespread *Plasmodium relictum* lineage GRW4 was accidentally introduced into the Hawaiian archipelago and, along with factors such as habitat destruction, urbanization and pollution, has led to a significant decline in native bird populations [1, 8]. This parasite lineage is closely related to the *P. relictum* lineage SGS1, which, although native to Europe, Africa and Asia [9], has also recently been reported as infecting wild birds in South America [10]. Thus, the spread of parasites *via* bird migration or natural dispersal to areas with competent insect vectors may help drive the distribution of avian malaria parasites [11]. In addition to natural mechanisms, the introduction of avian species into new areas by humans may also contribute to the spread of parasites infecting these birds. For example, Marzal et al. [12] described how house sparrows, one of the most widespread bird species in the world, have spread the parasites they harbour beyond their native range.

However, on the basis of molecular analyses of haemosporidian-infected birds captured in various places in Europe and Africa, it has been suggested that some parasite lineages are only transmitted in Africa, while others are only transmitted in Europe and a few in both continents [13]. The local circulation of parasites between resident and migratory species in Africa and the lack of circulation of these parasites in Europe [14] indicates that infected birds are less likely to complete migrations or that European hematophagous insects are not competent vectors for these lineages. In addition, geographical barriers may limit the distribution of parasite lineages; Mata et al. [15], for instance, found that the Strait of Gibraltar represents a natural barrier for blood-parasite dispersal between Europe and Africa, although the strength of the barrier effect varied among the parasite genera. While this may be the case with host-specialized malaria-like parasites of the genus *Haemoproteus*, it is possible that host-generalist lineages of *Plasmodium* are shared by wild birds on both continents.

Here, using results from previous studies, we provide strong evidence for local circulation in house sparrows from southern and central Spain of a haemosporidian lineage that had previously been thought to be restricted to Africa.

## Methods

### Study area and bird sampling

House sparrows were captured using mist-nets in 2012 and 2013 in two areas of the Iberian Peninsula. In central Spain (Badajoz Province), birds were sampled at three different locations (38°39′ N, 7°13′ W; 38°53′ N, 7°00′ W; and 38°55′ N, 6°58′ W) in March–April and infected individuals were kept in captivity to observe any temporal variation in the intensity of infection and their infection status in the context of different studies. In southern Spain, birds were collected at 45 different localities in the provinces of Huelva, Seville and Cadiz in July–October (see Ferraguti et al. [16] for further details of study areas). Both areas are characterized by a Mediterranean climate, with a long dry summer season and most precipitation concentrated in autumn and winter. Here, birds were ringed with numbered metal rings and immediately released unharmed at the site of capture after manipulation [17]. A blood sample was taken from the brachial (central Spain) or jugular (south Spain) veins of each bird using a sterile syringe. The volume of blood extracted depended on the size of the bird but never exceeded 1% of its body mass. In central Spain, samples were placed in Eppendorf tubes with SET buffer and stored at 4 °C until genomic DNA extraction. In southern Spain, samples were collected in Eppendorf tubes, maintained in cold boxes in the field and stored at 4 °C for 24 h prior to centrifugation for 10 min at 1700×*g* (4000× *rpm*) to separate serum and cellular fractions. The cellular fraction was then frozen at − 20 °C until subsequent molecular analysis.

### Molecular and phylogenetic analyses

Genomic DNA was extracted from samples from central and southern Spain using either a standard chloroform/isoamyl alcohol method [18] or the Maxwell^®^16 LEV system Research (Promega, Madison, WI, USA) [19]. A 478-bp fragment (excluding PCR primers) of the *Plasmodium* mitochondrial *cytochrome b* (*cytb*) gene was amplified following Hellgren et al. [20]. Negative samples were rescreened with the complete PCR protocol to avoid false negative samples as described by McClintock et al. [21]. Negative controls for both PCR reactions (at least one per plate) and DNA extraction (one per 15 samples) were included in the analysis.

Positive amplifications were sequenced using the Macrogen sequencing service (Macrogen Inc., Amsterdam, The Netherlands). The identity of each lineage was confirmed by sequenced bi-directionally with the complementary primer. Labelled DNA fragments of PCR positive products were sequenced with an ABI 3130xl automated sequencer (Applied Biosystems, Foster City, USA), using the same forward and reverse primers employed in the PCR reaction. Sequences were edited using the software Sequencher^®^ v.4.9 (Gene Codes Corp.,^®^ 1991–2009, Ann Arbor, MI, USA) and identified by assigning unknown *cytb* sequences to previously identified parasite lineages from GenBank and MalAvi databases [7].

The sequences of the 13 *Plasmodium* lineages detected during the study were edited and aligned using the software Geneious v1.8.0 [22]. The *Plasmodium* lineages isolated from house sparrows detected in southern Spain have previously been described in Ferraguti et al. [16]. Birds from central Spain were monitored during previous studies (e.g. Marzal et al. [12]) and these results are used here to discuss the local circulation of the lineage PAGRI02 in Europe. Phylogenetic trees were estimated with the Maximum Likelihood method using the software MEGA 7.0 and with an estimated N of bootstrap (*N *= 1000) [23]. Trees were visualized using Treeview [24]. A sequence of *Haemoproteus tartakovskyi* (subgenus *Parahaemoproteus*) was used as the outgroup.

## Results

**Fig. 1:**
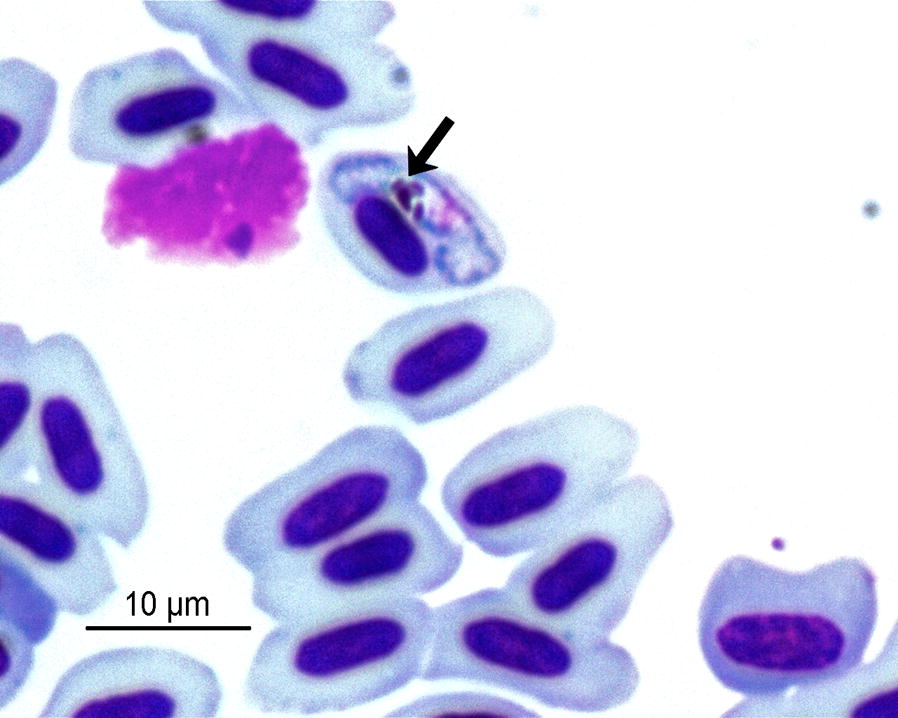
Blood parasites found in house sparrows from central Spain with details of *Plasmodium* sp. lineage PAGRI02. Arrow indicates the parasite cell

**Fig. 2:**
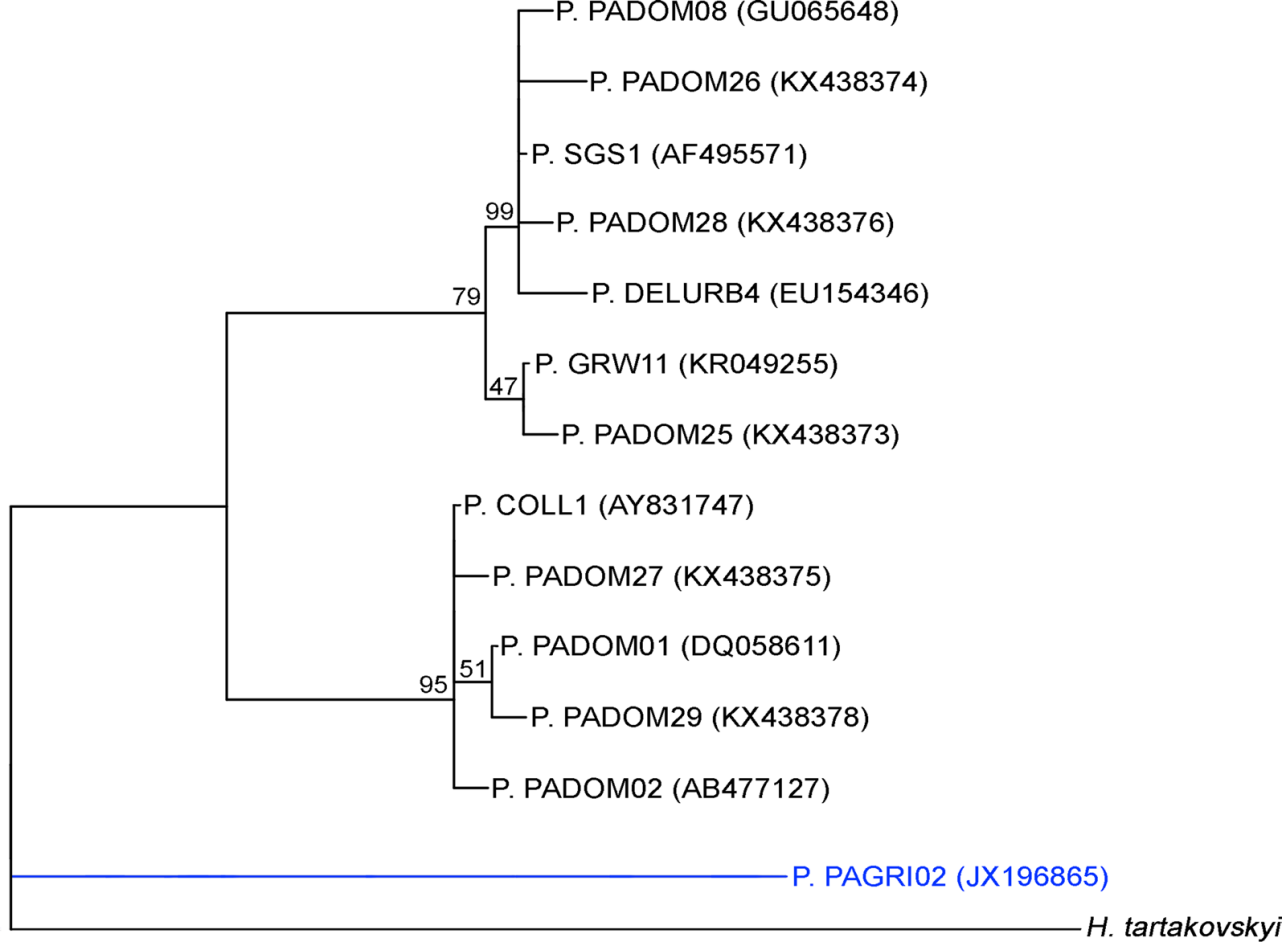
Phylogenetic Maximum Likelihood tree of haemosporidian lineages found infecting house sparrows (*Passer domesticus*) in this study. Sequences represent a fragment of 478 bp from the mtDNA *cytb* gene. Support values on nodes were calculated based on 1000 bootstrap repetitions. The *Plasmodium* lineage PAGRI02 is shown in blue. A sequence of *Haemoproteus tartakovskyi* (subgenus *Parahaemoproteus*) was used as the outgroup

Totals of 412 and 2588 house sparrows were sampled in central and southern Spain, respectively. Thirteen different lineages were identified; 86 and 760 birds were infected with 6 and 12 different *Plasmodium* lineages in central and southern Spain, respectively (Table 1). Eleven lineages were shared between the two regions. Specifically, the *Plasmodium* lineage PAGRI02 (Fig. 1) was found in five individuals: one adult male in a population from Badajoz and four birds (one adult male, two adult females and one female yearling) from four different localities in Huelva Province. The infection of the individual from central Spain was monitored for four months using methods including molecular techniques (nested PCRs and qPCRs) and the observation of blood smears throughout this period. The phylogenetic analysis of the *Plasmodium* sequences isolated in house sparrows from the study area revealed two main clades supported by high bootstrap (> 79) and that the PAGRI02 lineage did not cluster with any other lineages (Fig. 2). The PAGRI02 lineage differs by only 6 bp (99% identity) from the lineage PADOM16 belonging to *Plasmodium rouxi* (Additional file 1: Table S1), which suggests that both lineages correspond to the same morphospecies.

**Table 1:** Lineages found in house sparrows from this study with information on the countries and continents where they were previously reported according to the MalAvi database

Lineage	GenBank ID	Sample size	Country	Continent
COLL1	AY831747	2/103	Austria, Bulgaria, France, Portugal, Romania, South Africa, Spain, Tunisia	Africa, Europe
DELURB4	EU154346	0/1	Bulgaria, Hungary, Italy, Russia, Spain	Asia, Europe
GRW11	KR049255	16/80	Algeria, Armenia, Austria, Bulgaria, Czech Republic, France, Germany, Hungary, Israel, Italy, Japan, Lithuania, Morocco, Netherlands, Nigeria, Poland, Portugal, Romania, Russia, Serbia, South Africa, Spain, Sweden, Switzerland, Tunisia, Turkey, Ukraine, UK	Africa, Asia, Europe
PADOM01	DQ058611	19/27	Bermuda, Bulgaria, France, Italy, Portugal, Romania, Spain	Europe, Noth America
PADOM02	AB477127	0/23	China, Czech Republic, Egypt, France, Japan, South Korea, New Zealand, Norway, Romania, Russia, Spain, Turkey, USA	Africa, Asia, Europe, North America, Oceania
PADOM08	GU065648	1/0	Spain	Europe
PADOM25	KX438373	0/1	Spain	Europe
PADOM26	KX438374	0/1	Spain	Europe
PADOM27	KX438375	0/1	Spain	Europe
PADOM28	KX438376	0/2	Spain	Europe
PADOM29	KX438378	0/1	Spain	Europe
PAGRI02	JX196865	1/4	Bulgaria, Morocco, Nigeria, Spain, Tunisia	Africa, Europe
SGS1	AF495571	47/516	Algeria, Armenia, Austria, Belgium, Bulgaria, Canada, China, Czech Republic, Egypt, Falkland Islands, France, Germany, Hungary, India, Israel, Italy, Japan, Kenya, South Korea, Lithuania, Mongolia, Morocco, Netherlands, New Zealand, Nigeria, Norway, Peru, Poland, Portugal, Romania, Russia, Serbia, South Africa, Spain, Sweden, Switzerland, Tunisia, Turkey, Ukraine, UK	Africa, Asia, Europe, North America, Oceania, South America

*Notes*: The sample size column indicates the number of individuals found infected by each lineage in central/southern Spain. Lineages PADOM25 and PADOM26 are incorrectly labelled in MalAvi as *Haemoproteus* (accessed on 3 October 2019). The same accession numbers correspond to PADOM30 and PADOM31, which have the alternative names of PADOM25 and PADOM2

## Discussion

According to the MalAvi database (v.2.4.0, accessed 19 August 2019; [7]), 36 different *Plasmodium* lineages infect house sparrows worldwide and could play a role in the distribution and maintenance of parasites in both native and invaded areas [12, 25]. Here, we present strong evidence for the European circulation of the African *Plasmodium* lineage PAGRI02 in this widespread bird species. This result can be added to the findings of other studies indicating that generalist parasites disperse across continents *via* both migrant and invasive species [25]. Interestingly, this lineage was first found to infect African resident bird species such as *Passer griseus* captured in Nigeria (Bensch & Otosson, unpublished data) and house sparrows and Spanish sparrows (*Passer hispaniolensis*) in North Africa [26]. In addition, lineage PAGRI02 has been recently found infecting Spanish sparrows in Bulgaria (south-eastern Europe) [27], although this population is migratory and winters in North Africa [28]. In spite of the low prevalence of PAGRI02 in house sparrows in Spain, our study suggests that the area of transmission of this parasite lineage is broader than once previously thought and encompasses both Africa and Europe. Our results are based on the amplification of the barcoding region of avian malaria parasites (i.e. *cytb* gene). Thus, further molecular studies of additional independent loci [9, 29] are required to confirm whether the lineages found in Spain and those found in Africa form a monophyletic group.

The spread of haemosporidian parasites into new areas or hosts requires the presence of haemosporidian-infected birds, suitable vectors in which the haemosporidian parasites can complete their sexual cycle and susceptible host species that can be infected [1]. Although the migration of an infected bird from North Africa to southern Spain is possible, it is very unlikely that the birds were infected in Africa given that most *Passer domesticus* are residents in Europe and that lineage PAGRI02 was found in several birds from a number of different localities. Thus, PAGRI02 is probably transmitted in Spain by competent vectors. Previous studies suggest that mosquitoes have generalist relationships with *Plasmodium* parasites [30, 31] and that the different lineages of parasites infecting house sparrows are effectively transmitted by mosquitoes in the area [32]. Mosquitoes (Culicidae) of the genus *Culex* are the most suitable vectors for avian *Plasmodium* parasites [1]. In the study area, house sparrows are typical hosts of numerous mosquito species including *Cx. pipiens* and *Cx*. *perexiguus* [33, 34]. Although current information on the potential vectors of avian *Plasmodium* is scarce, molecular studies have isolated *Plasmodium* lineages from these mosquito species collected in the area [31, 34], thereby providing further evidence for their role in the local transmission of *Plasmodium* lineages [32, 35]. However, further experimental studies on the competence of native mosquito species for transmitting *Plasmodium* lineage PAGRI02 are required as the molecular detection of parasite DNA does not necessarily imply vector competence [36].

A number of alternatives exist to explain the presence of PAGRI02 *Plasmodium* in the Iberian Peninsula. First, the long-distance dispersal of infected insect vectors may overcome geographical barriers between Africa and Europe. It has been shown that wind can promote geographical shifts and range expansion in arthropod species and vector-borne diseases (e.g. *Culicoides* and bluetongue disease) between Africa and southern Europe [37, 38]. Additionally, vectors can be transported aboard ships or aeroplanes and thus may permit the spread of the parasite to remote locations. For example, it is presumed that the avian malaria vector *Culex quinquefasciatus* was accidentally introduced to Hawaii by the vessel HMS Wellington and has probably led to population declines in many native Hawaiian bird species [8, 39]. Secondly, infected *Passer* individuals could move from North Africa to Europe ([40], see also [28]) and then go on to spread the parasite lineage to different populations. For example, Spanish sparrows have been reported crossing the Strait of Gibraltar in both directions [41, 42]. Additionally, although house sparrows are considered as residents [43], some individuals in northern Norway dispersed from their native island and settled on a neighbouring island at distances of 2–20 km [44]. Moreover, an experimental study showed that adult house sparrows could move distances of 11–14 km [45]. These two dispersal distances are similar to the 14.4 km that separate Europe from Africa across the Strait of Gibraltar. Surprisingly, lineage PAGRI02 was detected in Badajoz in 2011 in two European pond turtles (*Mauremys leprosa*) (100% identity for a 452-bp sequence; A. Marzal, unpublished data). Alternatively, house sparrows infected with PAGRI02 may also be accidentally transported on boats or ships from Africa to Europe, as was the case of many introductions from Eurasia to Africa and the Americas in the 19th century [46, 47]. Thirdly, an infected *Passer griseus* may move from Africa and disperse into Spain. However, there is little evidence for this alternative as, to our knowledge, this species has never been reported from Spain [48]. Finally, an unidentified migratory species could have transported the parasite between continents, which then finally ended up by infecting resident *Passer* species inhabiting these areas [13]. Whatever the direct cause of the dispersion, our data confirm that lineage PAGRI02 can complete its reproductive cycle in Spain.

## Conclusions

Regardless of the method of dispersal, this study provides strong evidence for the active circulation in Europe of a *Plasmodium* lineage previously thought to circulate only in Africa. Unfortunately, we do not have any historical information about the lineages that were circulating in the area in the previous century that would help determine whether PAGRI02 was already present in Spain or has experienced a recent range expansion due to, for example, changes in environmental conditions. This result highlights the fact that the classification of *Plasmodium* lineages as either African- or European-transmitted lineages is not that straightforward, above all in the light of geographically extensive samplings. Understanding how environmental variables influence the distribution of parasite lineages is a key issue in disease ecology, especially since anthropogenic changes in climate and landscape are now affecting the distribution and incidence of vector-borne pathogens [49]. Temperature and precipitation are key variables explaining the prevalence of avian haemosporidians [50, 51]. This is especially relevant under a scenario of global change as an increase in temperature may provide new opportunities for avian malaria transmission in areas where the parasites were previous absent, thereby altering the current distribution of parasite lineages [52, 53]. Further studies on the global distribution of parasite lineages infecting birds should be conducted to identify the current transmission areas of these parasites.

## Supplementary information

**Additional file 1: Table S1.** *Plasmodium* lineages found infecting house sparrows (*P. domesticus*) in this study. The information of the putative morphospecies following the identity criterium according to GenBank is shown. For each lineage, the closest phylogenetically related lineage with known morphospecies is provided, reporting the fragment similarity (number of identical base pairs/sequence size).

## Data Availability

The data supporting the conclusions of this article are included within the article. The datasets used and/or analysed during the present study will be made available by the corresponding author upon reasonable request. Sequences used in this study are accessible in the GenBank database and the accession numbers reported in Table 1.

## Abbreviation

*cytb*: cytochrome *b* gene
