# Supplementary material for: From Africa to Europe: evidence of transmission of a tropical Plasmodium lineage in Spanish populations of house sparrows
Source: Parasit Vectors. 2019 Nov 21;12:548. doi: 10.1186/s13071-019-3804-1 (PMC6873688; doi:10.1186/s13071-019-3804-1)
Supplement: Supplementary file 1 — Additional file 1: Table S1. Plasmodium lineages found infecting house sparrows (P. domesticus) in this study. The information of the putative morphospecies following the identity criterium according to GenBank is shown. For each lineage, the closest phylogenetically related lineage with known morphospecies is provided, reporting the fragment similarity (number of identical base pairs/sequence size). [file 13071_2019_3804_MOESM1_ESM.docx]

**Additional file 1: Table S1.** *Plasmodium* lineages (GenBank accession numbers) found infecting house sparrows (*Passer domesticus*) in this study. The information of the putative morphospecies following the identity criterium according to GenBank is shown. For each lineage, the closest phylogenetically related lineage with known morphospecies is provided, reporting the fragment similarity (number of identical base pairs / sequence size).

| **Lineage** | **GenBank ID** | **Putative Morphospecies – Genetic Lineage**  **(base pair similarity)** |
| --- | --- | --- |
| COLL1 | AY831747 | *Plasmodium cathemerium* - SEIAUR01 (474/477) |
| DELURB4 | EU154346 | *Plasmodium relictum -* SGS1 (477/479) |
| GRW11 | KR049255 | *P. relictum -* GRW11 (478/478) |
| PADOM01 | DQ058611 | *P. cathemerium -* SEIAUR01 (473/477) |
| PADOM02 | AB477127 | *P. cathemerium* - SEIAUR01 (475/477) |
| PADOM08 | GU065648 | *P. relictum -* SGS1 (474/475) |
| PADOM25 | KX438373 | *P. relictum* - GRW11 (477/478) |
| PADOM26 | KX438374 | *P. relictum -* SGS1 (476/478) |
| PADOM27 | KX438375 | *P. cathemerium* - SEIAUR01 (472/476) |
| PADOM28 | KX438376 | *P. relictum* - SGS1 (477/478) |
| PADOM29 | KX438378 | *P. cathemerium* - SEIAUR01 (473/476) |
| PAGRI02 | JX196865 | *Plasmodium rouxi* - PADOM16 (467/473) |
| SGS1 | AF495571 | *P. relictum -* SGS1 (478/478) |
